# Supplementary material for: The neural dynamics of positive and negative expectations of pain
Source: eLife. 2024 Dec 2;13:RP97793. doi: 10.7554/eLife.97793 (PMC11616993; doi:10.7554/eLife.97793)
Supplement: Supplementary file 1. — (a) Common effects of positive and negative expectations compared to control in the anticipation phase. (b) Differential activation for expectation compared to neutral expectation in the pain phase. (c) Differential activation between placebo and nocebo in the anticipation phase. (d) Activation for positive expectations compared to negative expectations in the pain phase. (e) Combined EEG-fMRI analysis. [file elife-97793-supp1.docx]

**Supplementary File 1a**

*Peak coordinates and statistics of regions that showed common effects of positive and negative expectations compared to control in the anticipation phase*

| **Region** |  | **MNI Coordinates** | | |  |  |
| --- | --- | --- | --- | --- | --- | --- |
|  | **Hemi.** | **X** | **Y** | **Z** | ***t*** | ***p_FWE_*** |
| *Expectation > Neutral Expectation* | |  |  |  |  |  |
| Angular Gyrus | R | 56 | -54 | 40 | 5.92 | <.001 |
| Superior Frontal Gyrus | R | 8 | 44 | 36 | 5.57 | .002 |
|  | L | -4 | 30 | 62 | 4.88 | .049 |
| Insular Cortex | L | -28 | 22 | -6 | 5.33 | .006 |
| Paracingulate Gyrus | R | 6 | 46 | 12 | 5.02 | .027 |
| vmPFC | R | 14 | 56 | -14 | 5.00 | .029 |
| Anterior Cingulate Cortex | R | 4 | 42 | 12 | 4.89 | .047 |
|  | L | -2 | 40 | -4 | 3.96 | .025† |
| DLPFC | R | 40 | 24 | 36 | 4.44 | .004† |
|  | L | -32 | 18 | 36 | 3.93 | .026† |
| Thalamus | L | -6 | -12 | 4 | 3.91 | .038† |
| *Neutral Expectation > Expectation* | |  |  |  |  |  |
| Superior Parietal Lobule, Postcentral Gyrus | R | 34 | -36 | 44 | 5.62 | .001 |
| DLPFC | R | 30 | 2 | 62 | 3.74 | .048 |

*Note.* Coordinates are in MNI space. DLPFC = Dorsolateral Prefrontal Cortex. vmPFC = ventromedial prefrontal cortex. † small-volume corrected.

**Supplementary File 1b**

*Peak coordinates and statistics of regions that showed differential activation for expectation compared to neutral expectation in the pain phase*

| **Region** |  | **MNI Coordinates** | | |  |  |
| --- | --- | --- | --- | --- | --- | --- |
|  | **Hemi.** | **X** | **Y** | **Z** | ***t*** | ***p_FWE_*** |
| *Expectation > Neutral Expectation* | |  |  |  |  |  |
| Brain Stem | L | -14 | -28 | -30 | 4.14 | .026† |
| Thalamus | R | 4 | -4 | -2 | 4.05 | .023† |
| *Neutral Expectation > Expectation* | |  |  |  |  |  |
| Precentral Gyrus | R | 24 | -6 | 44 | 5.16 | .014 |
|  | L | -60 | -2 | 24 | 5.05 | .023 |
| Amygdala | L | -18 | 0 | -20 | 4.28 | .003† |
| Hippocampus | R | 32 | -22 | -14 | 4.19 | .009† |
| Insular Cortex | R | 46 | -10 | 16 | 4.01 | .041† |
| DLPFC | R | 42 | 10 | 24 | 3.82 | .037† |

*Note.* Coordinates are in MNI space. DLPFC = Dorsolateral Prefrontal Cortex. † small-volume corrected.

**Supplementary** **File 1c**

*Peak coordinates and statistics of regions that showed differential activation between placebo and nocebo in the anticipation phase*

| **Region** |  | **MNI Coordinates** | | |  |  |
| --- | --- | --- | --- | --- | --- | --- |
|  | **Hemi.** | **X** | **Y** | **Z** | ***t*** | ***p_FWE_*** |
| *Placebo > Nocebo* |  |  |  |  |  |  |
| Lingual Gyrus | L | -14 | -76 | -6 | 11.34 | <.001 |
| Occipital Fusiform Gyrus | L | -20 | -68 | -12 | 9.21 | <.001 |
| Precuneus | L | -2 | -62 | 16 | 5.42 | .004 |
| Supracalcarine Cortex | R | 14 | -62 | 16 | 5.05 | .024 |
| Superior LOC | L | -16 | -84 | 24 | 4.96 | .034 |
| Amygdala | R | 22 | 2 | -22 | 3.62 | .031† |
| *Nocebo > Placebo* |  |  |  |  |  |  |
| Lingual Gyrus | R | 10 | -80 | -6 | 13.27 | <.001 |
| LOC | R | 28 | -78 | 20 | 5.46 | .003 |
| Cerebellum VI | R | 34 | -62 | -20 | 5.09 | .019 |

*Note.* Coordinates are in MNI space. LOC = Lateral Occipital Cortex. † small-volume corrected.

**Supplementary File 1d**

*Peak coordinates and statistics of regions that showed larger activation for positive expectations compared to negative expectations in the pain phase*

| **Region** |  | **MNI Coordinates** | | |  |  |
| --- | --- | --- | --- | --- | --- | --- |
|  | **Hemi.** | **X** | **Y** | **Z** | ***t*** | ***p_FWE_*** |
| *Placebo > Nocebo* |  |  |  |  |  |  |
| Anterior SMG | L | -36 | -40 | 36 | 6.53 | <.001 |
| DLPFC | R | 40 | 14 | 46 | 4.80 | .001† |
|  | L | -22 | 8 | 44 | 6.29 | <.001† |
| Superior Parietal Lobule | R | 30 | -64 | 46 | 6.17 | <.001 |
|  | L | -30 | -60 | 44 | 5.16 | .014 |
| Middle Frontal Gyrus | L | -40 | 12 | 36 | 6.14 | <.001 |
| Angular Gyrus | R | 44 | -50 | 38 | 6.14 | <.001 |
| vmPFC | R | 42 | 46 | 10 | 5.72 | .001 |
|  | L | -42 | 50 | 2 | 6.00 | <.001 |
| Precuneus | R | 4 | -66 | 48 | 5.49 | .003 |
|  | L | -20 | -66 | 56 | 5.70 | .001 |
| Posterior SMG | R | 42 | -36 | 48 | 5.24 | .010 |
| Superior Frontal Gyrus | L | -24 | 6 | 56 | 5.19 | .012 |
| Middle Temporal Gyrus | R | 60 | -50 | -10 | 5.17 | .014 |
| Brain Stem | R | 4 | -42 | -38 | 4.06 | .035† |
|  | L | -2 | -42 | -34 | 5.16 | .014 |
| Inferior Frontal Gyrus | R | 44 | 12 | 18 | 4.89 | .047 |
|  | L | -44 | 34 | 14 | 4.97 | .033 |
| Anterior STG | L | -60 | -10 | 2 | 4.88 | .048 |
| Hippocampus | R | 28 | -16 | -18 | 4.81 | .001† |
|  | L | -24 | -18 | -16 | 3.82 | .032† |
| Central Operculum | L | -60 | -10 | 8 | 4.19 | .021† |
| Insula | L | -40 | -2 | 2 | 3.96 | .049† |
| Amygdala | R | 20 | -8 | -16 | 3.80 | .017† |
|  | L | -30 | -2 | -24 | 3.63 | .030† |
| *Nocebo > Placebo* |  |  |  |  |  |  |
| Thalamus | R | 6 | -6 | 2 | 3.90 | .048† |

*Note.* Coordinates are in MNI space. SMG = Supramarginal Gyrus. DLPFC = Dorsolateral Prefrontal Cortex. STG = Superior Temporal Gyrus. vmPFC = ventromedial prefrontal cortex. † small-volume corrected.

**Supplementary File 1e**

*Peak values of effects found in the combined EEG-fMRI analysis*

| **Region** |  |  | **Peak** | | | |
| --- | --- | --- | --- | --- | --- | --- |
|  | **Hemi.** | **Direction** | **Elec.** | **Freq.** | **Time** | ***t(40)*** |
| Anterior Insula | L | negative | F8 | 16 Hz | -3.0 s | -5.13 |
| Anterior Cingulate Cortex | L | positive | PO3 | 128 Hz | -2.1 s | 5.34 |
|  |  | positive | TP10 | 128 Hz | 0.2 s | 4.89 |
|  | R | positive | FT7 | 76.11 Hz | -0.1 s | 5.35 |
| DLPFC | L | negative | F5 | 26.89 Hz | -5.2 s | -5.47 |

**Note.** Elec = Electrode. Freq = Frequency. DLPFC = Dorsolateral Prefrontal Cortex. All peak *t*-values were significant at *p*< .001.
